# Predictors of Outcomes in Patients With Clinically Suspected Myocarditis: Analysis From a Single Center Registry

**DOI:** 10.31083/RCM48707

**Published:** 2026-06-26

**Authors:** Gassan Moady, Elia Yanko, Lihi Levi-Gofman, Dana Grosbard, Shaul Atar

**Affiliations:** ^1^Azrieli Faculty of Medicine, Bar Ilan University, 1311502 Safed, Israel; ^2^Department of Cardiology, Galilee Medical Center, 22100 Nahariya, Israel

**Keywords:** acute myocarditis, ejection fraction, outcome, echocardiography

## Abstract

**Background:**

Acute myocarditis is an inflammatory condition of the myocardium that typically follows a benign course, although fulminant cases can be fatal. The clinical trajectory is largely determined by the degree of left ventricular ejection fraction (LVEF) impairment and the presence of associated complications. Although LVEF often recovers within weeks to months after the acute episode, some patients develop persistent dysfunction. This study aimed to identify potential predictors of outcomes following acute myocarditis using data from a single-center registry.

**Methods:**

This retrospective study included 157 patients with clinically suspected myocarditis. Demographic, echocardiographic, and laboratory variables, as well as clinical outcome data, including recurrent readmissions and persistent cardiac dysfunction, were collected. Associations between echocardiographic and laboratory parameters and outcomes were analyzed.

**Results:**

Approximately 30% of patients had reduced LVEF (LVEF <55%) during the index hospitalization. Among patients with reduced LVEF, 53% exhibited persistent cardiac dysfunction at 4–6 weeks of follow-up. Patients with reduced LVEF had higher troponin and N-terminal pro-B-type natriuretic peptide levels, as well as longer hospital length of stay. Readmission rates for chest pain were comparable between groups. Reduced LVEF during the index hospitalization and diabetes were the strongest independent predictors of persistent cardiac dysfunction.

**Conclusions:**

Cardiac dysfunction is common in clinically suspected myocarditis but is not associated with increased readmission rates. Reduced LVEF and diabetes emerged as potential predictors of persistent cardiac dysfunction following the acute episode.

## 1. Introduction

Acute myocarditis is an inflammatory disorder of the myocardium, most commonly developing after viral infection [1]. The reported incidence ranges from 10 to 100 cases per 100,000 individuals annually worldwide; however, the true prevalence is likely underestimated because of non-specific clinical manifestations and frequent underdiagnosis [2]. In clinical practice, definitive diagnosis based on established criteria such as endomyocardial biopsy (EMB) is not routinely performed. Instead, many patients are classified as having clinically suspected myocarditis based on clinical presentation, laboratory findings, electrocardiographic abnormalities, and echocardiographic features [3]. Historically, EMB was regarded as the diagnostic gold standard, enabling immunohistological evaluation and molecular detection of microbial genomes within myocardial tissue [4]. However, its invasive nature, procedural risks, and limited sensitivity, combined with the increasing availability of cardiac magnetic resonance (CMR), have restricted its use to selected cases [4,5,6]. Advanced CMR techniques, including T1 and T2 mapping and extracellular volume quantification, are now widely employed to differentiate acute from chronic myocardial inflammation [6,7]. By definition, acute myocarditis is associated with elevated troponin levels, reflecting myocardial injury, although normal values may still be observed in a subset of patients [8,9,10]. Additional biomarkers such as natriuretic peptides, short non-coding RNAs, fibrinogen-to-albumin ratio, and soluble interleukin-2 receptor have also been investigated for diagnostic and prognostic purposes [11,12,13,14]. Management is generally supportive and symptom-directed in mild cases without left ventricular ejection fraction (LVEF) impairment or heart failure (HF) symptoms, whereas severe presentations with cardiogenic shock or refractory ventricular arrhythmias may require mechanical circulatory support [15,16]. Although most patients experience complete recovery of cardiac function after the acute phase, up to 25% may develop persistent stable dysfunction, and 10%–25% may progress to worsening ventricular impairment [1]. The present study aimed to describe outcomes in a single-center cohort of patients with clinically suspected myocarditis and to identify clinical and echocardiographic parameters predictive of persistent cardiac dysfunction.

## 2. Methods

### 2.1 Study Population

The study population comprised hospitalized patients diagnosed with acute myocarditis at Galilee Medical Center in the cardiology and intensive cardiac care units during the years 2019–2023. Patients were retrospectively identified using the diagnostic terms “acute myocarditis”, “acute idiopathic myocarditis”, and “myocarditis, unspecified”. The diagnosis was established on the basis of a compatible clinical presentation (e.g., chest pain, dyspnea, palpitations, or syncope), elevated and dynamic troponin levels, and echocardiographic evidence of global left ventricular dysfunction, with or without pericardial effusion. In selected cases, CMR imaging was performed during the index hospitalization or within several weeks after discharge to further support the diagnosis. Invasive coronary angiography (ICA) was undertaken in patients with clinical features suggestive of myocardial infarction to exclude obstructive coronary artery disease. In-hospital management was individualized according to disease severity. Patients with mild symptoms were treated with antipyretics and analgesics, whereas oral or intravenous diuretics were administered in cases of volume overload, particularly when pulmonary congestion was evident. Beta-blockers and renin–angiotensin–aldosterone system (RAAS) inhibitors were initiated in patients with left ventricular dysfunction accompanied by clinical signs of heart failure.

### 2.2 Echocardiography

All patients underwent transthoracic echocardiography using a Philips EPIQ 7 system with an EPIQ X8-2t transducer (Philips, Andover, MA, USA). Echocardiographic measurements were acquired and interpreted by a cardiologist. LVEF was calculated using Simpson’s biplane method from apical four- and two-chamber views, according to the formula: LVEF = [(LV end-diastolic volume – LV end-systolic volume) / LV end-diastolic volume] × 100. Left ventricular end-diastolic diameter (LVEDD) was obtained in the parasternal long-axis view. Reduced LVEF was defined as below 55%, and persistent cardiac dysfunction as LVEF <55% in the follow up echocardiography. Diastolic function was assessed by calculating the E/eʹ ratio, derived from the average septal and lateral mitral annular tissue Doppler velocities. Left atrial volume index (LAVI) was determined at end-ventricular systole and calculated as LAVI = left atrial volume / body surface area. In patients with reduced LVEF during the index hospitalization, follow-up echocardiography was performed 4–6 weeks later.

### 2.3 ECG and Laboratory Findings

Electrocardiographic (ECG) recordings were obtained for all patients upon admission. Pathological findings were defined as ST-segment depression 80 ms after the J-point or new T-wave inversion at the nadir. Atrial and ventricular arrhythmias were also documented during hospitalization. High-sensitivity troponin I (hs-TnI) levels were measured using the ARCHITECT c-TnI assay (Abbott Park, IL, USA), with abnormal values defined as >20 ng/L in women and >30 ng/L in men. Additional laboratory parameters included C-reactive protein (CRP), hemoglobin, white blood cell (WBC) count, and renal function tests.

### 2.4 Outcomes

Clinical outcomes assessed were length of hospital stay (LOS), in-hospital complications including arrhythmia and HF symptoms, and readmissions for chest pain or HF symptoms. Echocardiographic outcomes included evaluation of LVEF during the index hospitalization and at 4–6 weeks of follow-up in cases where systolic function was reduced.

### 2.5 Statistical Analysis

Qualitative variables are presented as percentages, while quantitative variables are expressed as means with standard deviation (SD) or medians with interquartile ranges (IQR). Spearman’s rank correlation test was applied to assess the association between laboratory values and LOS. The Wilcoxon rank-sum test was used to examine associations between clinical parameters and the occurrence of complications. Multiple regression analysis was performed to identify predictors of persistent cardiac dysfunction. A two-sided alpha level of 0.05 was considered statistically significant. A sample size of 49 patients was required to achieve 80% statistical power. Statistical analyses were conducted using IBM SPSS Statistics 27 software (Version 27, IBM Corp., Armonk, NY, USA). The study was approved by the local ethics committee of Galilee Medical Center; approval number NHR-0121-23.

## 3. Results

**Fig. 1.**
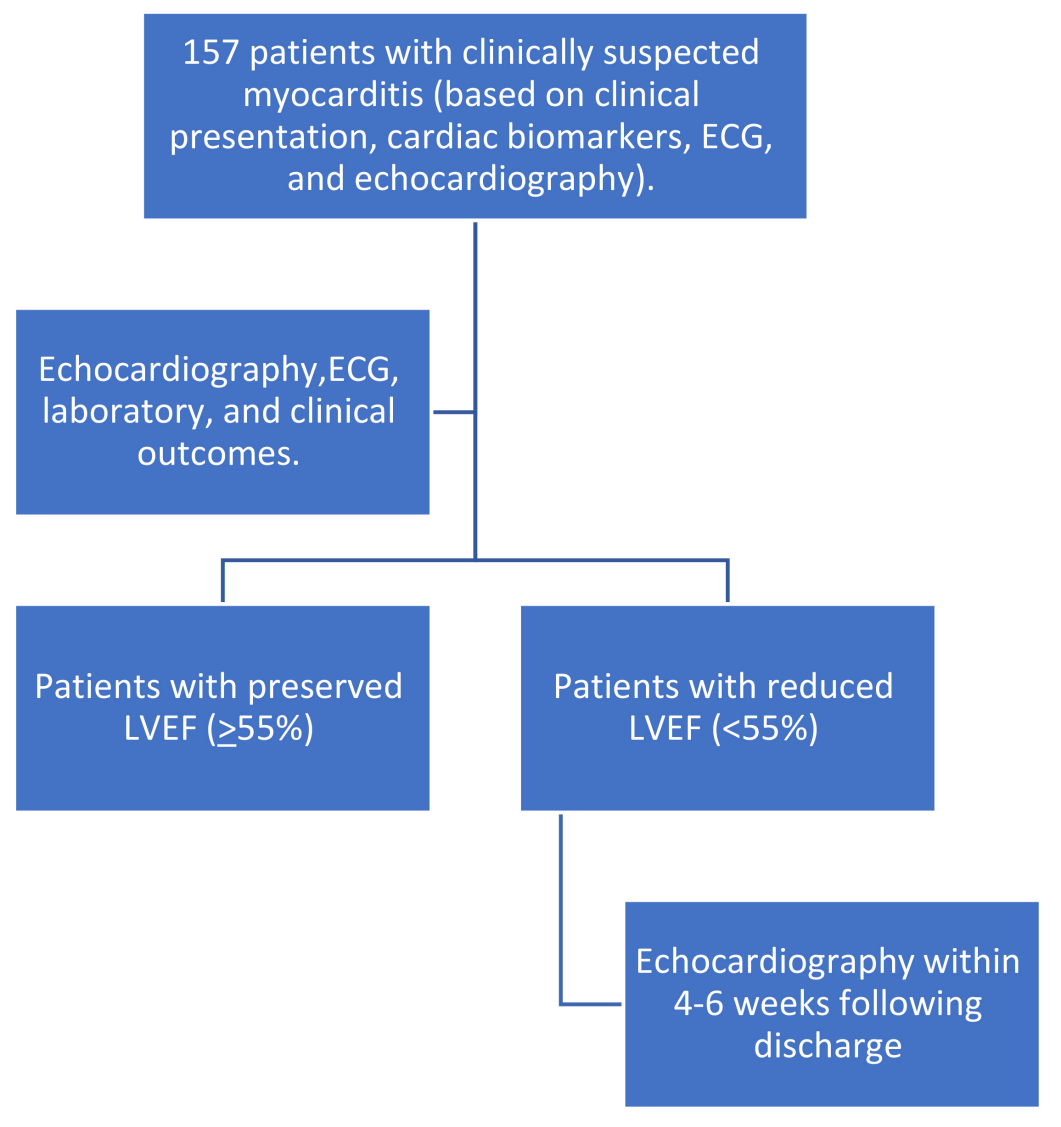
**Study flowchart**. ECG, electrocardiogram; LVEF, left ventricular ejection fraction.

A total of 157 patients (median age 30 years, 82% males) were included in the final analysis. The flowchart of the study is depicted in Fig. 1.

Only 3% of patients had a prior history of coronary artery disease (CAD), and none had a history of myocarditis before the index hospitalization. The predominant presenting symptom was chest pain, followed by palpitations, dyspnea, and syncope. Baseline comorbidities and medication use in the study population are summarized in Table 1.

**Table 1.** **Baseline characteristics of the patients**.

n		157
Age, median (IQR)		30 [22, 40]
Male, n (%)		129 (82)
Hypertension, n (%)		12 (8)
CAD, n (%)		5 (3)
Hyperlipidemia, n (%)		38 (24)
Diabetes mellitus, n (%)		11 (7)
CKD, n (%)		26 (16)
Tobacco use, n (%)		75 (48)
Presenting symptom		
	Chest pain, n (%)	115 (73)
	Palpitations, n (%)	23 (15)
	Dyspnea, n (%)	17 (11)
	Syncope, n (%)	2 (1)
Previous medications		
	Statins, n (%)	67 (43)
	Aspirin, n (%)	38 (24)
	Anti-hypertensive, n (%)	10 (6)
	Anti-glycemic, n (%)	11 (7)

CAD, coronary artery disease; CKD, chronic kidney disease (defined as estimated glomerular filtration rate <60 mL/min/1.73 m^2^); IQR, interquartile range.

Upon presentation, mean systolic blood pressure (SBP) was 120 mmHg and mean LVEF was 55%, indicating that most patients were hemodynamically stable. During the hospital stay, 16% of patients were treated with intravenous diuretics to alleviate congestion symptoms based on clinical examination and chest X-ray. Patients with reduced cardiac function (defined as LVEF <55%) were treated with beta blockers and RAAS inhibitors. Table 2 summarizes the laboratory, echocardiographic, and main outcomes during the index hospitalization.

**Table 2.** **Hemodynamic, laboratory, echocardiographic, and disease course of the study population**.

n		157
SBP (mmHg), mean ± SD		120 ± 14
HR (BPM), mean ± SD		76 ± 12
ECG changes, n (%)		60 (38)
WBC (×10^3^/µL), Mean ± SD		10 ± 4
Creatinine (mg/dL), Mean ± SD		0.9 ± 0.3
Hemoglobin (g/dL), Mean ± SD		14 ± 1.5
CRP (mg/L), Median [IQR]		54 [25, 131]
Troponin (ng/L), Median [IQR]		3069 [604, 8748]
CMR, n (%)		47 (30)
Coronary angiography, n (%)		28 (18)
Echocardiographic parameters		
	LVEF (%), mean ± SD	55 ± 9
	LVEDD (mm), mean ± SD	47 ± 5
	LAVI (mL/m^2^), mean ± SD	24 ± 6
	E/e', mean ± SD	9 ± 2
Therapy during hospitalization		
	Diuretics, n (%)	25 (16)
	Beta blockers, n (%)	40 (25)
	RAAS inhibitors, n (%)	30 (19)
LOS (days), Median [IQR]		4 [3, 5]
Complications, n (%)		35 (22)

BPM, beats per minute; CMR, cardiac magnetic resonance; CRP, C-reactive protein; HR, heart rate; LAVI, left atrial volume index; LOS, length of stay; LVEDD, left ventricular end diastolic diameter; LVEF, left ventricular ejection fraction; RAAS, renin angiotensin aldosterone system; SBP, systolic blood pressure; SD, standard deviation; WBC, white blood cell.

In a subset of patients (30%), CMR results were available. In them, the findings were consistent with myocarditis. In about 50% of them, high T2 signal was documented, whereas late gadolinium enhancement (LGE) in all of them (mainly subepicardial or mid-wall).

**Fig. 2.**
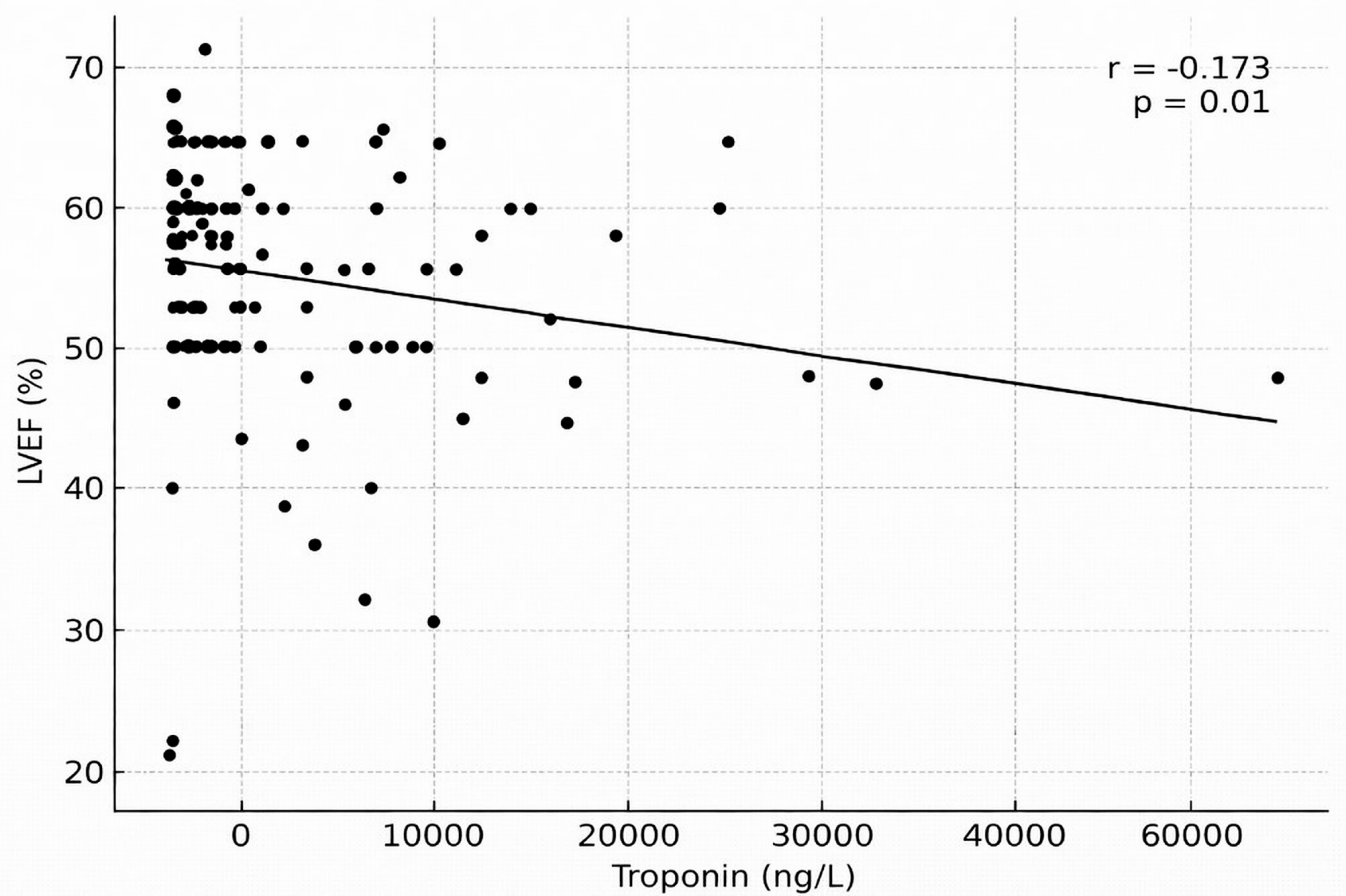
**Correlation between troponin level and LVEF. **An inverse weak correlation exists between troponin level and LVEF. Troponin level usually reflects the degree of inflammation and tissue edema. However, this correlation is not linear as some patients with severe cases of fulminant myocarditis with extensive LGE may have modest troponin elevations, and *vice versa*. LGE, late gadolinium enhancement.

High troponin levels were correlated to reduced LVEF as shown in Fig. 2.

**Fig. 3.**
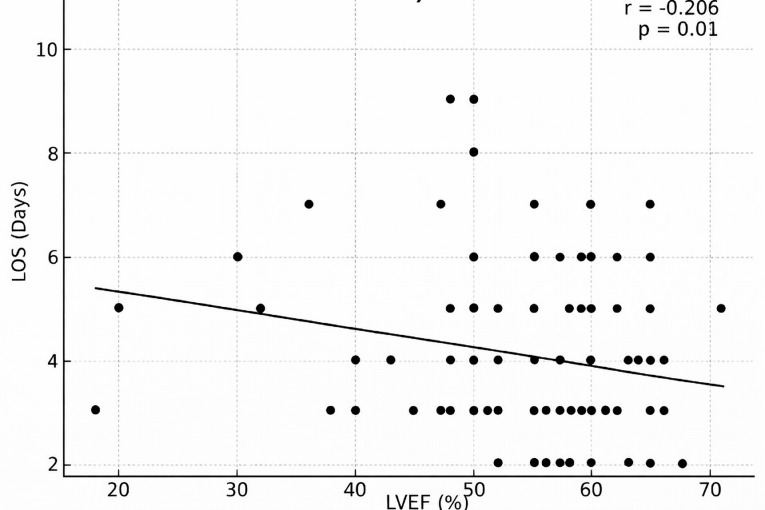
**Correlation between LVEF and LOS. **A weak correlation (r = –0.206) is seen between LVEF and LOS. LOS is largely affected by several factors and therefore may not always reflect the severity of the disease.

Reduced LVEF was weakly correlated to longer LOS (r = –0.206) as illustrated in Fig. 3.

**Fig. 4.**
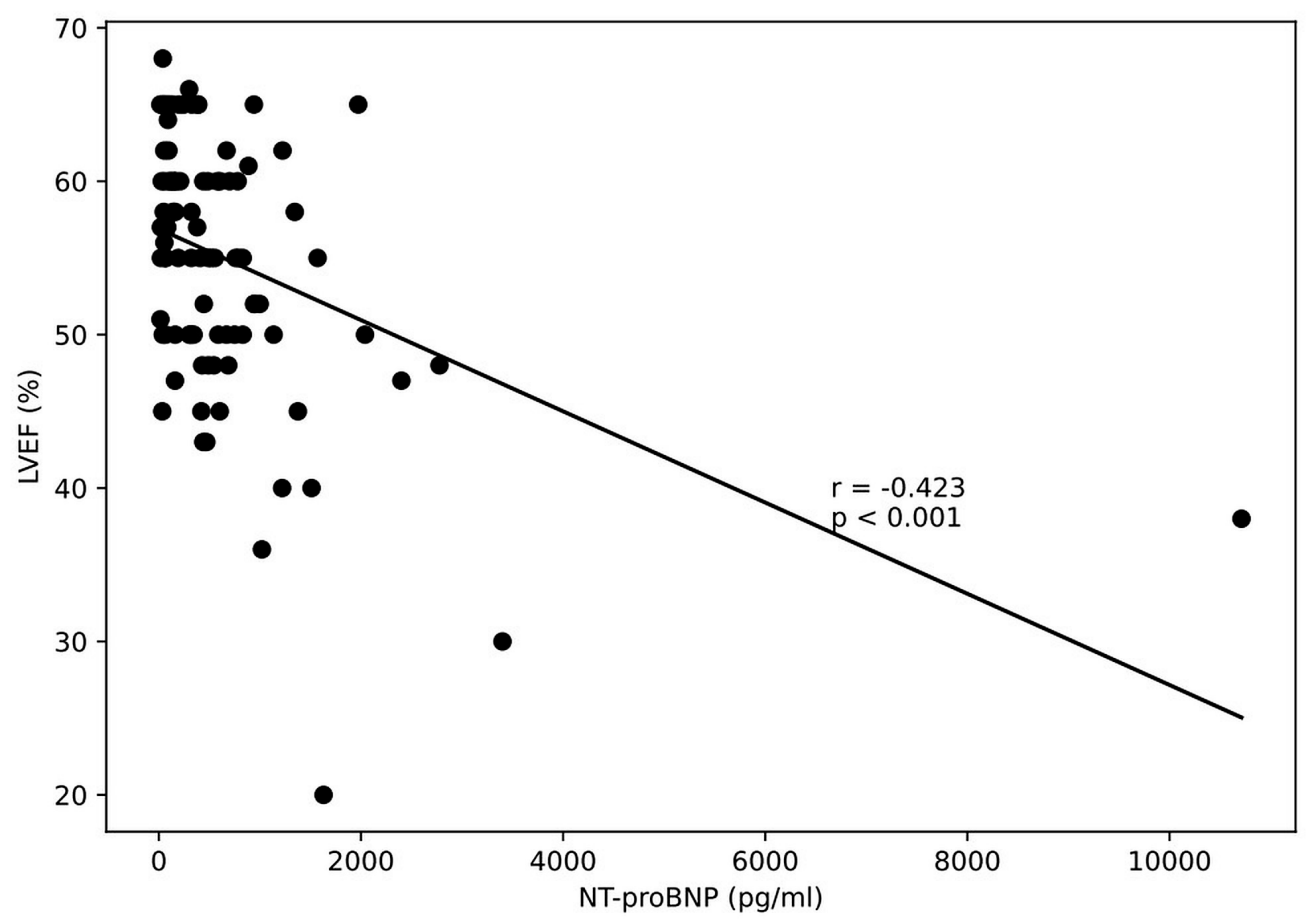
**Correlation between NT-proBNP and LVEF**. A modest inverse correlation is demonstrated between NT-proBNP levels and LVEF (r = –0.423). The degree of wall stress reflected by the level of NT-proBNP correlates with tissue inflammation and edema, and thereby with the degree of cardiac dysfunction. NT-proBNP, N-terminal prohormone of brain natriuretic peptide.

In 98 (62%) patients, N-terminal prohormone of brain natriuretic peptide (NT-proBNP) levels were available. NT-proBNP was inversely correlated with LVEF (r = –0.423, *p* < 0.001). Scatter plot is depicted in Fig. 4.

**Fig. 5.**
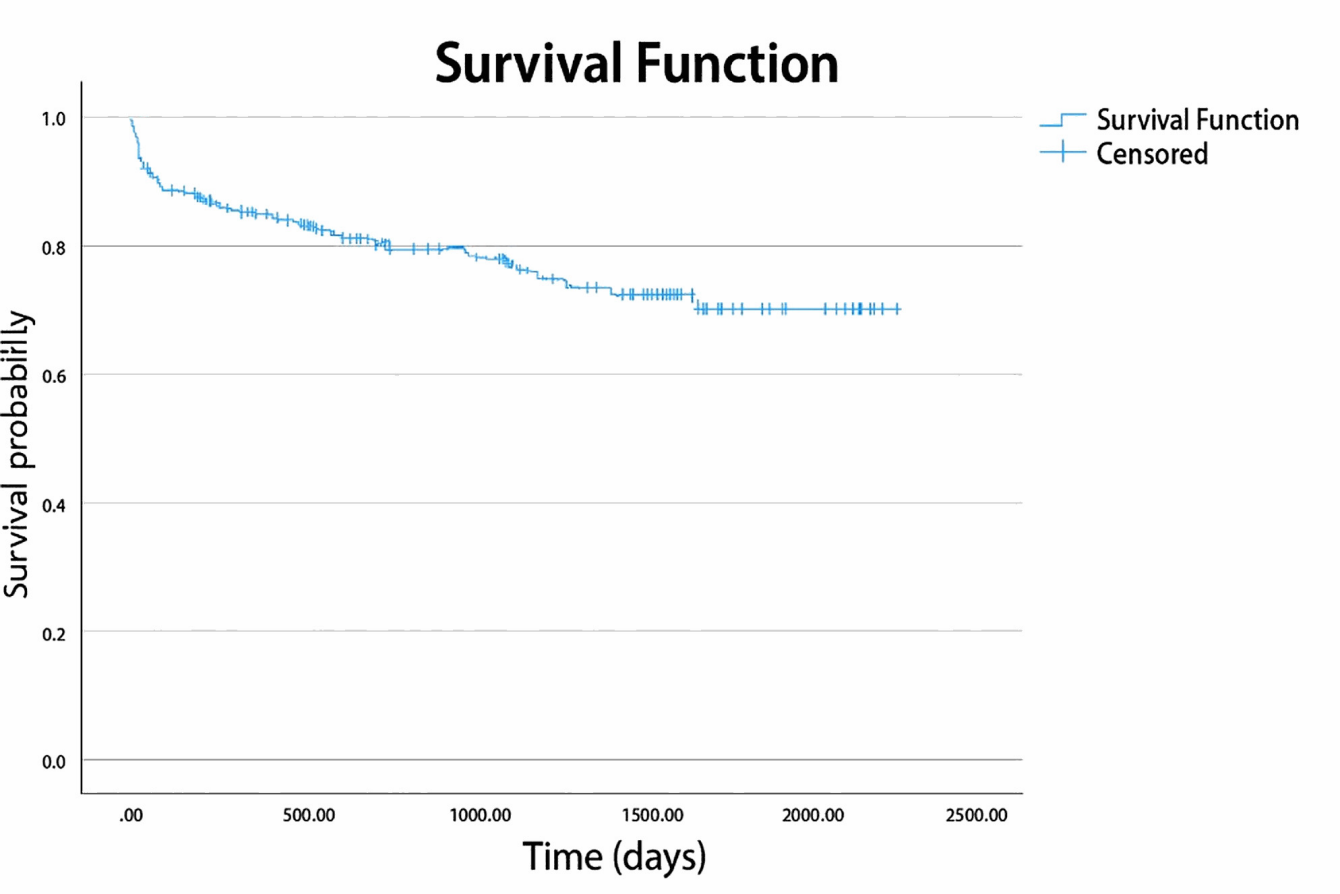
**Kaplan-Meier curve for readmissions**.

Using Kaplan-Meier curve we report the readmissions for recurrent chest pain among patients as depicted in Fig. 5.

**Fig. 6.**
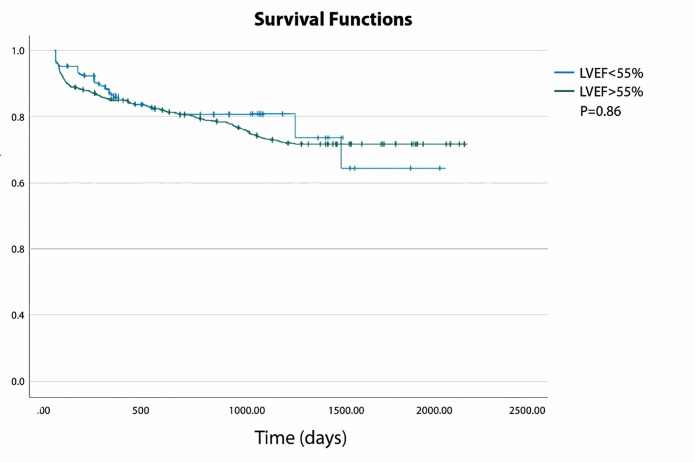
**Readmission-free survival stratified by LVEF group**.

During the index hospitalization, 47 patients (30%) had reduced LVEF (defined as LVEF <55%). The rate of readmission for chest pain or HF symptoms was similar between patients with reduced and preserved LVEF [OR = 0.94; 95% CI, 0.45–1.94; *p* = 0.86]. The Kaplan–Meier curve illustrating readmissions by LVEF group is shown in Fig. 6.

During follow-up, patients with reduced LVEF underwent repeat echocardiography after 4–6 weeks. Among them, 25 (53%) still had reduced LVEF. All patients with reduced LVEF were discharged on beta-blockers and RAAS inhibitors. Of note, diastolic function was normalized in all patients. Multiple regression analysis was conducted in those with persistent ventricular dysfunction to identify potential predictors of incomplete recovery. Table 3 summarizes the results.

**Table 3.** **Multiple logistic regression for predicting persistent reduced LVEF**.

	OR	95% CI	*p* value
Age	1.09	0.90–1.20	0.056
Reduced LVEF	2.03	1.42–2.90	<0.001
CRP levels	1.01	0.99–1.02	0.131
Diabetes	10.01	1.30–10.10	0.038
Gender	1.04	0.09–12.26	0.974
Hyperlipidemia	0.36	0.04–3.00	0.345
Hypertension	0.69	0.01–43.59	0.859
Tobacco	0.37	0.06–2.43	0.303
Troponin levels	1.00	1.00–1.00	0.273

After adjusting to potential confounders, reduced LVEF and diabetes remain significant predictors for persistent reduced LVEF. It is noteworthy that a history of diabetes independently increased this risk by tenfold (OR = 10.01, *p* = 0.038), making it an important factor for identifying high-risk patients in clinical practice. Baseline LVEF also represents an important risk factor. The lower the LVEF during the index event, the greater the risk for persistent cardiac dysfunction.

## 4. Discussion

In this study, we evaluated 157 patients with clinically suspected myocarditis and found that 30% exhibited reduced LVEF during the acute phase. Approximately half of these patients achieved complete echocardiographic recovery (LVEF >55%) within four to six weeks following the initial episode. Among the evaluated variables, reduced LVEF in the index event and diabetes emerged as potential predictors for persistent cardiac dysfunction. Patients with reduced LVEF in the index event were not at increased risk for readmissions in our study. Possible explanation for this finding may be that our cohort included stable patients with relatively mild reduction in LVEF. Also, biomarkers reflecting “acute injury” such as troponin may not be reliable for long term prognostication, while long-term remodeling depends more on structural changes or associated conditions such as diabetes. Diabetes was previously identified as a risk modifier in myocarditis that may increase the risk for HF [17]. The inflammatory process in myocarditis may be enhanced in diabetic patients leading to delayed healing, increased myocardial injury, and maladaptive remodeling [17]. The extent of LGE on CMR imaging and the initial clinical presentation also represent important prognostic indicators for recovery [16]. In a multicenter registry of 684 patients with acute myocarditis, individuals presenting with LVEF <50%, ventricular arrhythmias, or low cardiac output syndrome were at significantly higher risk compared with uncomplicated cases, which generally had a benign course and a low likelihood of subsequent left ventricular systolic dysfunction [15]. Similarly, in another cohort of patients with clinically suspected myocarditis, advanced New York Heart Association functional class, immunohistological evidence of myocardial inflammation, and lack of beta-blocker therapy were associated with adverse outcomes [18]. Fulminant myocarditis and other complicated forms of the disease are also more frequently linked to persistently reduced LVEF over the long term than non-fulminant or uncomplicated cases [19,20]. In a recent international cohort of fulminant myocarditis, older age, lower arterial pH, and a histopathologic diagnosis of giant cell myocarditis independently predicted in-hospital and one-year mortality [21]. In our cohort, diastolic dysfunction normalized in all evaluated patients. Diastolic impairment is commonly observed during the acute phase, likely related to myocardial inflammation and edema; in the subacute and chronic phases, residual edema and evolving fibrosis may contribute to increased stiffness and impaired relaxation [22]. In mild myocarditis, diastolic dysfunction typically resolves within weeks, whereas in severe cases it may persist due to extensive fibrosis. In the absence of significant tissue injury, diastolic function is generally preserved. In the present study, approximately half of the patients with reduced LVEF demonstrated recovery within one month after discharge. All patients were hemodynamically stable at discharge and did not exhibit heart failure symptoms. Those with reduced LVEF were discharged on beta-blockers and RAAS inhibitors. Because the clinical condition can deteriorate rapidly, sometimes within hours, with progression to cardiogenic shock or malignant arrhythmias, hospitalization for close monitoring is essential in all patients with suspected myocarditis. Risk stratification should integrate clinical findings, laboratory data, and multimodality imaging approaches [23,24]. Among 210 patients with endomyocardial biopsy-proven myocarditis followed for two years at Charité Hospital (Berlin, Germany) under standard heart failure therapy, approximately half achieved normalization of LVEF, while the remainder exhibited persistent systolic dysfunction [8]. It is important to note that in our study, follow-up echocardiography was performed only within a few weeks after discharge; longer-term follow-up would likely reveal a higher proportion of patients achieving complete recovery. Early initiation of medications for HF at discharge is essential to facilitate ventricular remodeling and relieve symptoms [8]. A persistent clinical dilemma in acute myocarditis management is whether to withdraw therapy after LVEF normalization. Treatment decisions should be guided by clinical judgment, echocardiographic findings, and cardiac magnetic resonance imaging. Notably, patients with a substantial burden of LGE may benefit from continued beta-blocker and RAAS inhibitors to reduce the risk of ventricular arrhythmias and limit further cardiac remodeling. Diabetic patients may benefit from early reassessment of cardiac function and potential long-term of anti-remodeling agents.

## 5. Limitations

Our study has several limitations. First, due to the retrospective design, there are several potential confounders that may influence the outcomes and were not controlled and thereby, our findings should be interpreted as associative rather than causal. Second, the majority of patients were diagnosed based on clinical presentation, biomarkers, and echocardiography findings. In selected patients, CMR was performed and confirmed the diagnosis while ICA was performed to exclude CAD in patients with suspected acute coronary syndrome (ACS). Therefore, most patients were classified as clinically suspected myocarditis, and our findings should be projected to this population. In clinical practice, many young patients are diagnosed based on these criteria particularly if they are in stable condition. Third, patients were hemodynamically stable, and therefore, conclusions may not be applicable for other severe cases of myocarditis. Fourth, we assessed the subgroup of patients with reduced LVEF within approximately one month after discharge. This may lead to an overestimation of the recovery rate (as the recovery process has not yet been completed). The recommended period to reevaluate cardiac function under medical therapy is usually three to six months. A relatively high percentage of patients (about 50%) had already complete remission in cardiac function within weeks. Cardiac function remission depends on baseline function, the amount of LGE in CMR, and the time in which anti remodeling agents are initiated.

## 6. Conclusions

In mild cases of clinically suspected myocarditis, transient impairment of cardiac function is common but does not appear to be associated with an increased rate of readmissions for chest pain or HF symptoms. Diabetes and reduced LVEF at the index event emerge as the strongest predictors for persistent LV dysfunction. Early initiation of HF-directed medical therapy is essential to promote rapid and complete recovery of ventricular function.

## Data Availability

Study data will be available upon reasonable request from the corresponding author (GM).
